# Strategies for successful trauma registry implementation in low- and middle-income countries—protocol for a systematic review

**DOI:** 10.1186/s13643-018-0700-2

**Published:** 2018-02-21

**Authors:** Tiffany Paradis, Etienne St-Louis, Tara Landry, Dan Poenaru

**Affiliations:** 1 McGill University, 3655 Promenade Sir William Osler, Montreal, QC H3A 1A3 Canada; 2 McGill University Health Centre, 1001 Decarie Boulevard, Montreal, QC H4A 3J1 Canada

**Keywords:** Trauma, Trauma registries, Low-middle-income countries, Database, Trauma information, Acute injury data, Resource-limited setting

## Abstract

**Background:**

The benefits of trauma registries have been well described. The crucial data they provide may guide injury prevention strategies, inform resource allocation, and support advocacy and policy. This has been shown to reduce trauma-related mortality in various settings. Trauma remains a leading cause of mortality in low- and middle-income countries (LMICs). However, the implementation of trauma registries in LMICs can be challenging due to lack of funding, specialized personnel, and infrastructure. This study explores strategies for successful trauma registry implementation in LMICs.

**Methods:**

The protocol was registered a priori (CRD42017058586). A peer-reviewed search strategy of multiple databases will be developed with a senior librarian. As per PRISMA guidelines, first screen of references based on abstract and title and subsequent full-text review will be conducted by two independent reviewers. Disagreements that cannot be resolved by discussion between reviewers shall be arbitrated by the principal investigator. Data extraction will be performed using a pre-defined data extraction sheet. Finally, bibliographies of included articles will be hand-searched. Studies of any design will be included if they describe or review development and implementation of a trauma registry in LMICs. No language or period restrictions will be applied. Summary statistics and qualitative meta-narrative analyses will be performed.

**Discussion:**

The significant burden of trauma in LMIC environments presents unique challenges and limitations. Adapted strategies for deployment and maintenance of sustainable trauma registries are needed. Our methodology will systematically identify recommendations and strategies for successful trauma registry implementation in LMICs and describe threats and barriers to this endeavor.

**Systematic review registration:**

The protocol was registered on the PROSPERO international prospective register of systematic reviews (CRD42017058586).

**Electronic supplementary material:**

The online version of this article (10.1186/s13643-018-0700-2) contains supplementary material, which is available to authorized users.

## Background

A trauma registry is a clinical database that captures information on large patient cohorts in order to analyze the epidemiology, study the processes, and evaluate the quality of patient care in trauma [1]. Trauma registries vary widely in their content, administration, and cost. In high-income countries (HICs), trauma registry maintenance has been reported to have an estimated direct cost of approximately USD $95 per patient in 2015 [2]. This can be explained by the large infrastructure and resource investments required to operate expansive trauma registries such as the National Trauma Data Bank (NTDB) in the USA or other national and provincial trauma registries such as the Quebec Trauma Registry or the Australian Trauma Registry [3–7].

The variables within different trauma registries may differ in various contexts; however, the majority include data pertaining to pre-hospital care, in-hospital interventions, injury classification, physiological response (e.g., vital signs, laboratory data), complications, and patient outcomes [1].

Trauma registries have multiple uses such as injury surveillance, clinical research, and outcomes benchmarking. Trauma data are instrumental in designing targeted quality improvement initiatives, planning resource allocation, understanding pre-hospital care and transport priorities, and tracking changes in trauma system performance over time. They have enabled the reorganization of trauma delivery into more efficient regional systems of trauma care and have played a critical role in the dramatic improvements in trauma mortality observed in many HICs [8].

Injuries cause over five million deaths per year in low- and middle-income countries (LMICs); trauma is the primary cause of mortality and morbidity in persons aged 5 to 44 years old [9, 10]. The burden of trauma mortality disproportionately affects persons living in low-income countries. For example, road traffic injuries caused 2.03% of all deaths in low-income countries in 2015, nearly double the 1.07% rate for the same year in high-income countries [11]. Therefore, development of locally relevant and sustainable trauma registries in LMICs is a public health priority. Many LMICs lack the necessary infrastructure to implement or maintain costly trauma registries similar to those operating in HICs. A scoping review of the literature published by O’Reilly and colleagues specifically investigated the distribution of trauma registry publications by continent, by country, and by United Nations Development Index (UNDI) grouping [12]. They reported that a vast majority of trauma registry publications came from HICs, while only 1% came from the lowest UNDI grouping. Nevertheless, their study documented a proliferation of trauma registries in LMICs in the last decade [12]. They found that trauma registries in LMICs typically collect fewer variables, specifically with respect to processes of care and in-hospital management. They also usually have less stringent inclusion criteria, such that all injured patients are typically entered in the registry. On the other hand, HIC trauma registries tend to collect a much larger number of variables for a more restrictive group of patients, typically those with a greater severity of injury or those requiring prolonged hospitalization [12].

Trauma registry implementation requires a precisely defined population, adequately trained personnel, a dependable system of data collection, and the ability to analyze, report, and validate the data in a useful way [13]. Achieving these steps requires sufficient funding, which is often dependent on buy-in from stakeholders including health authorities. Financial limitations, scarcity of equipment and specialized workforce, lack of adequate health care policies and legislations, and increased trauma burden render the implementation of trauma registries very challenging in LMICs [14]. Where trauma registries do exist, they are often incomplete, subject to significant backlog, or simply cease their operations [15]. In resource-limited settings, health care dollars must be invested judiciously—consequently, trauma registries in such settings should be well-adapted to their context and must not incur prohibitive costs [8].

Improvements in health care and policy have been observed in developing countries with established trauma registries. For example, the pediatric trauma care in a large Nigerian teaching hospital was reorganized following the deployment of a simple low-cost trauma registry [16]. Another simplified trauma dataset was launched in Uganda, allowing the establishment of the Kampala Trauma Score as a triage tool in low-resource settings where high-tech measures of injury severity are not achievable [17].

No existing literature provides recommendations or guidelines for successful trauma registry implementation in a resource-limited setting. Our aim is to systematically review reports of trauma registry implementation in LMICs and to identify key parameters that contribute to success as well as factors that impede the successful implementation of a trauma registry.

## Methods

The protocol was registered a priori in the PROSPERO international prospective register of systematic reviews (CRD42017058586).

A peer-reviewed search strategy was developed in collaboration with a senior hospital librarian (TL) (Additional file 1). It will be used to search the following databases for relevant studies: MEDLINE (via Ovid, 1946 to 20/Feb/2017; via PubMed, 1946 to 20/Feb/2017), Embase (via Ovid, 1947 to 20/Feb/2017), Biosis Previews (via Ovid, 1969 to 2017 week 12), Global Health (via Ovid, 1973 to 2017 week 06), Africa-Wide Information (via Ebsco), the Database of Abstracts of Reviews of Effects (via The Cochrane Library, to issue 2 of 4 April 2015), the CENTRAL Registry of Controlled Trials (via The Cochrane Library, to issue 1 of 12, January 2017), The Cochrane Methodology Register (via The Cochrane Library, to issue 3 of 4, July 2012), the NHS Economic Evaluation Database (to issue 2 of 4, April 2015), LILACS (via Bireme), ProQuest Dissertations & Theses Global (via ProQuest), Scopus (via Elsevier), and Web of Science (via ThomsonReuters). The search strategy used text words and relevant indexing to identify articles discussing trauma registries in low- or low-middle-income countries. The full MEDLINE strategy (see Additional file 1) was applied to all databases, with modifications to search terms as necessary.

Adhering to PRISMA recommendations [18], two independent reviewers (TP, ESL) will perform a first screening based on title and abstract review. Disagreements not resolved by discussion between the reviewers shall be arbitrated by the principal investigator (DP). After first screen, the remaining studies will undergo full-text review and data extraction by the same independent reviewers. Data extraction will be performed using a pre-defined data extraction sheet (Additional file 2). It includes study location, authorship, HIC partnership, funding, age of registry, scope of cases covered, number of facilities, dedicated data collectors, registry administration modality, timing of data collection, constituent variables, duration of follow-up, and presence of data quality assurance mechanisms. Furthermore, comments and citations salient to the success factors and inhibitors will be recorded. Finally, the bibliography of included articles will be hand-searched. EndNote 8 Software (Clarivate Analytics, Philadelphia, PA) will be used to upload references, eliminate duplicates, and perform screening. The selection of studies will be conducted according to the eligibility criteria outlined in Table 1.

**Table 1:** Eligibility criteria presented in PICOS format

	Include	Exclude
Population	Any age Male and female	Not applicable
Intervention	Trauma registry design and/or development or implementation Trauma registry implementation/deployment Description of barriers and challenges to registry development or implementation	No description of design, development, implementation, or deployment Injury surveillance through other means than a trauma registry Narrow focus of registry (e.g., burns, traumatic brain injury)
Control	Not applicable	Not applicable
Outcome	Registry utilization and sustainability Registry data analysis	Comparative outcomes of pre-existing registries
Setting	Low-income country Lower middle-income country Middle-income country	High-income country

Summary and descriptive statistics will be reported in terms of means, standard deviations, medians, and ranges, as appropriate. As we will not be aggregating comparative or descriptive outcome data, meta-analysis is not appropriate. We will however synthesize the qualitative data in a systematic way through meta-narrative analysis, in accordance with the recommendations put forth by the Realist and Meta-Narrative Evidence Syntheses (RAMESES) project [19]. In so doing, the key qualitative findings from included studies will be presented with a specific focus on the key meta-narratives that are relevant to the successful implementation of trauma registries, and the commonalities and differences between them [20]. We suspect that qualitative data yielded from surveys, questionnaires, or interviews is likely to contribute significantly to our understanding of the challenges inherent to trauma registry implementation in low-resource settings. The PRISMA checklist is included in Additional file 3 [18]. The Methodological Index for Non-Randomized Studies (MINORS) instrument will be used to critically appraise the quantitative studies [21]. Qualitative studies, which are subject to different biases by virtue of their design and methods, serve a different purpose than quantitative studies and will not undergo risk of bias assessment. The quality of reporting for implementation of trauma registries will be assessed when applicable using a checklist from the Research Effectiveness Adoption Implementation and Maintenance (RE-AIM) Framework [22, 23].

The World Bank classification of countries by income was used to define low-income, lower middle-income, and middle-income countries [24]. This classification is based on gross national income (GNI) per capita. GNI per capita cut-offs for different income classes for the 2018 fiscal year are given in Table 2.

**Table 2:** Gross national income (GNI) per capita cut-off values for different income classes of the World Bank classification of economies in the fiscal year 2018

Group name	GNI per capita
Low-income economies	$0 to $1005
Lower middle-income economies	$1006 to $3955
Upper middle-income economies	$3956 to $12,235
Upper income economies	$12,236 or greater

## Discussion

We acknowledge several limitations to this study. Trauma registries themselves have limitations, particularly when they are hospital based, due to a selection bias introduced by virtue of the fact that many injured patients either cannot reach a hospital or do not seek medical treatment [25]. Furthermore, in institutions where baseline medical documentation is limited, it can be challenging to obtain a precise estimate of patient uptake within the registry. This can skew the interpretation of registry data by deflating the denominator [26]. Although this systematic review will adhere to PRISMA guidelines and follow strict methodological principles, it remains impossible to completely account for the limitations of the studies included within the review itself. For example, there may be significant heterogeneity in the studies as well as moderate to high risk of bias. These elements can decrease the validity of any results or conclusions of a systematic review. Furthermore, systematic reviews can be limited in their ability to identify all relevant negative or inconclusive studies due to publication bias. We attempted to overcome this by searching gray literature and conference proceedings, in addition to traditional databases of published literature.

Burdened by high clinical volumes, limited resources, lack of specialized workforce, and vulnerable populations, health care providers in LMICs face multiple barriers to effective and universal provision of health care [27]. Clinicians in the most resource-deprived parts of the world understand the need for quality improvement initiatives endorsed by health authorities and funded appropriately to make sustainable changes for better access to care and patient outcomes. However, these same clinicians may have few resources at their disposal to conduct high-yield research and implement impactful projects. Many efforts have been deployed to provide guidelines for optimization and prioritization of essential trauma care in LMICs [28, 29]. Similar LMIC-centered endeavors have been deployed in collaboration with HIC partners to define optimal resources for children’s surgery and define standards for research and quality improvement in resource-limited settings [30]. Given the significant burden of morbidity and mortality resulting from traumatic injury in children in LMICs, and acknowledging the importance of trauma systems that can collect, analyze, and synthesize data on injury mechanisms and outcomes, this study will investigate the successful factors for trauma registry implementation in LMICs, as well as its inhibitors.

Trauma registries vary widely in their patient inclusion criteria, in the extent of data they collect, and the funding they receive. This variability is not undesirable in-and-of itself, given the necessity for a local trauma registry to be adapted to the specific context in which it will be used. General strategies, recommendations, and precautions for successful trauma registry implementation can nevertheless be extracted from the growing published experience of trauma registry use in LMICs. To our knowledge, no study has yet reviewed and summarized the pearls and pitfalls of trauma registry implementation in LMICs. This methodology will provide trauma registry developers in LMICs with an evidence-based framework built on the experience of colleagues from around the world. This information will have the potential to improve local and international efforts to initiate and maintain trauma registries in LMICs.

## Additional files

Additional file 1: Search Strategy. Complete search strategy developed for the systematic review protocol. (PDF 297 kb)

Additional file 2: Data Extraction Sheet. Template that will be used to extract data from included articles in systematic review. (PDF 51 kb)

Additional file 3: PRISMA 2009 Checklist. Preferred reporting items for systematic review and meta-analysis checklist completed for the present protocol. (PDF 73 kb)

## Abbreviations

HICs: High-income countries
LMICs: Low- and middle-income countries
