# Supplementary material for: Strategies for successful trauma registry implementation in low- and middle-income countries—protocol for a systematic review
Source: Syst Rev. 2018 Feb 21;7:33. doi: 10.1186/s13643-018-0700-2 (PMC5822522; doi:10.1186/s13643-018-0700-2)
Supplement: Supplementary file 1 — Search Strategy. Complete search strategy developed for the systematic review protocol. (PDF 297 kb) [file 13643_2018_700_MOESM1_ESM.pdf]

Additional File 1. Search Strategy

Date:	*2017-02-23*
From:	*Tara Landry (MUHC - MGH library; 43057)*
To:	*Étienne St-Louis*
Subject:	*Trauma registries in LIC or LMIC countries*

1. Methodology 2
2. Searches 3
   2.1. Ovid Medline 3
   2.2. PubMed Medline 6
   2.3. Ovid Embase 11
   2.4. Africa-Wide Information 14
   2.5. Biosis Previews 17
   2.6. The Cochrane Library 19
   2.7. Global Health 20
   2.8. LILACS 22
   2.9. ProQuest Dissertations & Theses Global 22
   2.10. Scopus 23
   2.11. Web of Science 25

1. Methodology

The following databases were searched for relevant studies: MEDLINE (via Ovid, 1946 to 20/Feb/2017; via PubMed, 1946 to 20/Feb/2017); Embase (via Ovid, 1947 to 20/Feb/2017); Biosis Previews (via Ovid, 1969 to 2017 Week 12); Global Health (via Ovid, 1973 to 2017 Week 06); Africa-Wide Information (via Ebsco); the Database of Abstracts of Reviews of Effects (via The Cochrane Library, to issue 2 of 4 April 2015); the CENTRAL Registry of Controlled Trials (via The Cochrane Library, to issue 1 of 12, January 2017); The Cochrane Methodology Register (via The Cochrane Library, to issue 3 of 4, July 2012); the NHS Economic Evaluation Database (to issue 2 of 4, April 2015); LILACS (via Bireme); ProQuest Dissertations & Theses Global (via ProQuest); Scopus (via Elsevier) and Web of Science (via ThomsonReuters) were searched for relevant studies. The search strategy used text words and relevant indexing to identify articles discussing trauma registries in low- or low-middle income countries. The full MEDLINE strategy (see below) was applied to all databases, with modifications to search terms as necessary.

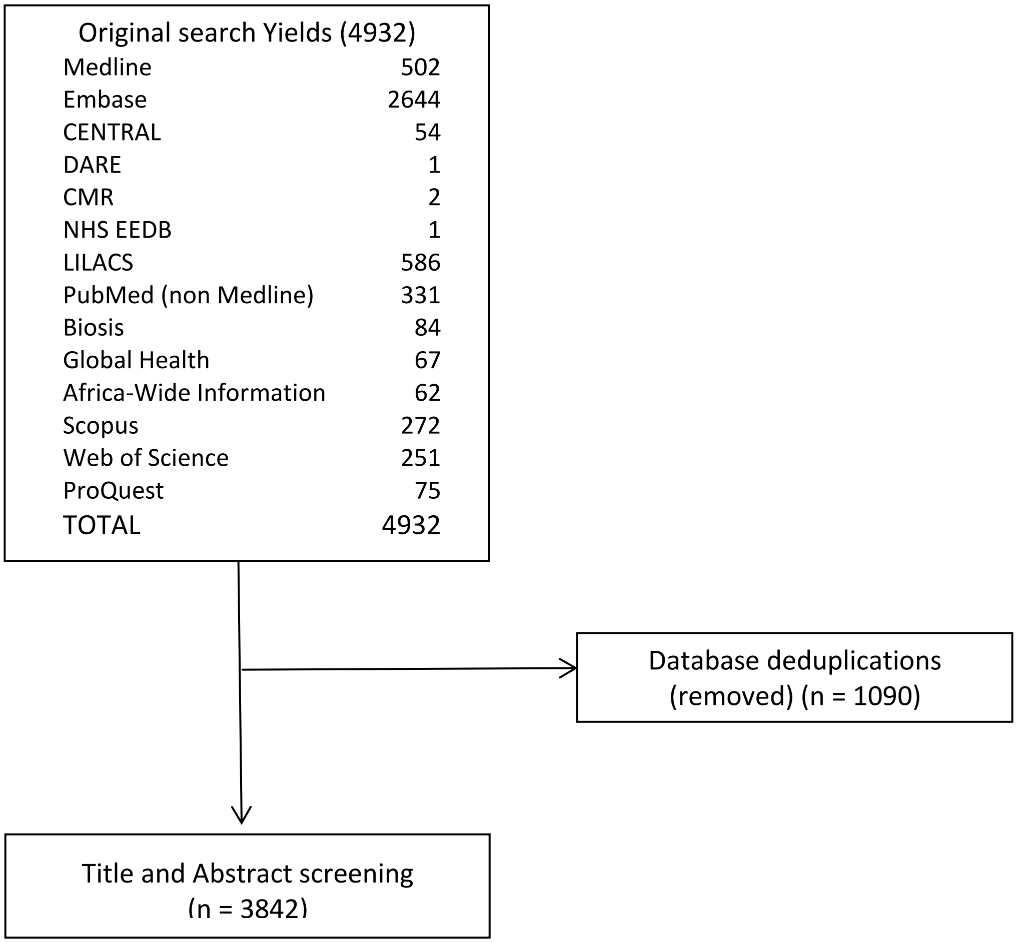

2. Searches

2.1. Ovid Medline

1 Registries/ (67061)
2 exp Databases as Topic/ (118214)
3 1 or 2 (181949)
4 exp "Wounds and Injuries"/ (802499)
5 exp Emergency Service, Hospital/ (60550)
6 4 or 5 (849674)
7 3 and 6 (8410)
8 (trauma adj2 (database* or data-base* or regist*)).tw,kf. (3704)
9 7 or 8 (10703)
10 Afghanistan/ or afghanistan.tw,kf,ia. (5527)
11 exp Africa, Eastern/ or (East* Africa or Burundi or Djibouti or Somaliland or Eritrea or Ethiopia or Abyssinia or Kenya or Rwanda or Ruanda or Somalia or Sudan or Tanzania or Tanganyika or Zanzibar or Urundi or Uganda or (Somali* adj1 (democratic or republic))).tw,kf,ia. (67640)
12 exp Africa, Western/ or (West*-Africa or Benin or Dahomey or Burkina Faso or Upper Volta or Cape verde or Cabo Verde or cap-vert or cote d'ivoire or ivory coast or Cote dilvoire or Gambia or Ghana or Gold Coast or Guinea or Liberia or Mali or Mauritania or Mauretania or Niger or Nigeria or Senegal or Senegambia or Sierra Leone or Togo or Togolese or Togoland or (sudan* adj1 republic)).tw,kf,ia. (182973)
13 Armenia/ or armenia*.tw,kf,ia. (2293)
14 Bangladesh/ or (Bangladesh or East-bengal).tw,kf,ia. (11687)
15 Bhutan/ or Bhutan.tw,kf,ia. (504)
16 Bolivia/ or boliv*.tw,kf,ia. (4218)
17 Cambodia/ or (cambodia or Kampuchea* or Khmer or kampudja).tw,kf,ia. (3861)
18 Cameroon/ or (cameroun or cameroon).tw,kf,ia. (6325)
19 Central African Republic/ or ("ubangi-shari" or Centrafrique or ((central-africa* or centrafrican or central african) adj2 (republic or empire))).tw,kf,ia. (1051)
20 Chad/ or (chad or tchad or tshad).tw,kf,ia. (1120)
21 Comoros/ or (comoros or comores or comoro or mayotte).tw,kf,ia. (517)
22 Congo/ or Democratic Republic of the Congo/ or (katanga or zaire or congo or leopoldville).tw,kf,ia. (12600)
23 Egypt/ or (egypt or "united arab republic").tw,kf,ia. (17517)
24 El Salvador/ or salvador.tw,kf,ia. (2871)
25 Guatemala/ or Guatemala.tw,kf,ia. (3706)
26 Haiti/ or (haiti or hispaniola).tw,kf,ia. (3590)
27 Honduras/ or honduras.tw,kf,ia. (1605)
28 exp India/ or (india or Sikkim).tw,kf,ia. (119303)
29 Indonesia/ or (indonesia* or Celebes or East Indies or Irian Jaya or Java or Madoera or Madura or Malay Archipelago or "Netherlands East Indies" or New Guinea West or Sulawesi or Sumatra or Timor or West Irian).tw,kf,ia. (16591)
30 Kashmir.tw,kf,ia. (1137)
31 (kiribati or nauru or tuvalu).tw,kf,ia. (272)
32 "Democratic People's Republic of Korea"/ or (North* Korea or (Korea adj5 demo*)).tw,kf,ia. (558)
33 Kosovo/ or kosovo.tw,kf,ia. (747)

34 Kyrgyzstan/ or (Kirghiz* or Kirgiz* or Kyrgyz*).tw,kf,ia. (1522)
35 Laos/ or (laos or lao pdr or (lao and democratic republic)).tw,kf,ia. (2422)
36 Lesotho/ or (lesotho or Basutoland).tw,kf,ia. (587)
37 Madagascar/ or (madagascar or malagasy).tw,kf,ia. (4535)
38 Malawi/ or (malawi or nyasaland).tw,kf,ia. (5697)
39 Micronesia/ or micronesia*.tw,kf,ia. (1549)
40 Moldova/ or (moldova* or Moldavia*).tw,kf,ia. (1029)
41 Mongolia/ or mongolia*.tw,kf,ia. (7477)
42 Morocco/ or (morocco or ifni).tw,kf,ia. (6317)
43 Mozambique/ or (mozambique or Mocambique or mozambic).tw,kf,ia. (2973)
44 Myanmar/ or (myanmar or burma).tw,kf,ia. (3056)
45 Nepal/ or Nepal.tw,kf,ia. (8419)
46 Nicaragua/ or nicaragua.tw,kf,ia. (1819)
47 Pakistan/ or Pakistan.tw,kf,ia. (18296)
48 Papua New Guinea/ or Papua.tw,kf,ia. (5266)
49 Philippines/ or (Philippines or Philipines or Phillipines or Phillippines or Mindanao or Luzon or Visayas).tw,kf,ia. (10194)
50 exp Samoa/ or samoa*.tw,kf,ia. (1216)
51 Sao Tome.tw,kf,ia. (203)
52 ((Caroline or Ellice or Gilbert or Johnston or Mariana or Marshall or Pacific or Solomon) adj1 (Island or islands)).tw,kf,ia. (3032)
53 Sri Lanka/ or (ceylon or sri lanka).tw,kf,ia. (6823)
54 Swaziland/ or swaziland.tw,kf,in. (804)
55 Syria/ or syria*.tw,kf,ia. (11054)
56 Tajikistan/ or (Tajikistan or Tadjik* or Tadzhik* or Tajik).tw,kf,ia. (929)
57 Timor-Leste/ or (Timor Leste or East Timor or Portuguese-Timor).tw,kf,ia. (379)
58 Tonga/ or tonga*.tw,kf,ia. (620)
59 Tunisia/ or (Tunisia or Tunis).tw,kf,ia. (8746)
60 Ukraine/ or ukrain*.tw,kf,ia. (17037)
61 Uzbekistan/ or uzbek*.tw,kf,ia. (2205)
62 Vanuatu/ or (vanuatu or new hebrides).tw,kf,ia. (594)
63 Vietnam/ or (vietnam or viet-nam).tw,kf,ia. (14909)
64 (west bank or gaza or palestin*).tw,kf,ia. (2713)
65 Yemen/ or (yemen* or aden or sanaa).tw,kf,ia. (2184)
66 Yugoslavia/ (9058)
67 limit 65 to yr="1994 - 2013" (2109)
68 (Yugoslavia or vojvodina).tw,kf,ia. (3073)
69 Zambia/ or zambia.tw,kf,ia. (5013)
70 Zimbabwe/ or (zimbabwe or rhodesia).tw,kf,ia. (6627)
71 10 or 11 or 12 or 13 or 14 or 15 or 16 or 17 or 18 or 19 or 20 or 21 or 22 or 23 or 24 or 25 or 26 or 27 or 28 or 29 or 30 or 31 or 32 or 33 or 34 or 35 or 36 or 37 or 38 or 39 or 40 or 41 or 42 or 43 or 44 or 45 or 46 or 47 or 48 or 49 or 50 or 51 or 52 or 53 or 54 or 55 or 56 or 57 or 58 or 59 or 60 or 61 or 62 or 63 or 64 or 67 or 68 or 69 or 70 (587231)
72 (kabul or Porto-Novo or Hogbonou or Adjace or Cotonou or Kutonu or Ouagadougou or Ouaga or Bujumbura or Usumbura or Phnom Penh or Bangui or Bangi or N'Djamena or Ndjamena or Fort Lamy or Moroni or Kinshasa or Asmara or Asmera or Addis Ababa or Addis Abeba or Banjul or Bathurst or Conakry or Bissau or Port-au-Prince or Pyongyang or Monrovia or Antananarivo or

Tananarive or Tana or Lilongwe or Bamako or Maputo or Lourenco Marques or Kathmandu or Niamey or Kigali or Freetown or Free-town or Mogadishu or Xamar or Hamar or Muqdisho or Maqadishu or Juba or Dodoma or Dar es Salaam or Lome or Kampala or Harare or Salisbury or Yerevan or Dhaka or Dacca or Thimphu or Thimbu or Sucre or Charcas or La Plata or Chuquisaca or La Paz or Praia or Yaounde or Jaunde or Brazzaville or Yamoussoukro or Cairo or Accra).tw,kf,ia. (22068)

73 (Tegucigalpa or Tegus or New Delhi or Jakarta or Nairobi or South Tarawa or Tarawa Teinainano or Pristina or Prishtina or Bishkek or Pishpek or Frunze or Vientiane or Maseru or Nouakchott or Palikir or Chisinau or Kishinev or Rabat or Nay Pyi Taw or Naypyidaw or Nepranytau or Naypyitaw or Kyetpyay or Pyinmana or Kyatpyay or Pyinmana or Yangon or Rangoon or Managua or Abuja or Lagos or Islamabad or Port Moresby or Moresby or Pom Town or Manila or Apia or Dakar or Honiara or Jayawardenepura or Jayewardenepura or Khartoum or Mbabane or Embabane or Lobamba or Damascus or Dushanbe or Dyushambe or Stalinabad or Dili or Kyiv or Kiev or Tashkent or Toshkent or Port Vila or Hanoi or Ha Noi or Sana'a or Sanaa or Sana or Lusaka or Ulaanbaatar or Ulan-Bator or Nuku-Alofa).tw,kf,ia. (19172)

74 72 or 73 (41000)

75 Developing Countries/ (68116)

76 (LMIC or LMICs or LAMIC or LAMICs or ((deprived* or underserved or under-served or transitional or poor or underdevel* or devel* or low* or less or least or under or mid* or third) adj3 (countr* or nation or econom*))).tw,kf. (152122)

77 75 or 76 (179125)

78 71 or 74 or 77 (721130)

79 9 and 77 (510)

80 remove duplicates from 78 (502)

Tara Landry, MLIS
MUHC Montreal General Hospital Medical Library
tara.landry@muhc.mcgill.ca
http://www.muhclibraries.ca/

2.2. PubMed Medline

Search	Query	Items found
#78	Search (#76) AND #77	331
#77	Search publisher[sb] NOT pmcbook	502081
#76	Search (#3) AND #75	12836
#75	Search ((#68) OR #71) OR #74	1167787
#74	Search (#72) OR #73	547109
#73	Search ((deprived*[Text Word] OR underserved[Text Word] OR under-served[Text Word] OR transitional[Text Word] OR poor[Text Word] OR underdevel*[Text Word] OR devel*[Text Word] OR low[Text Word] OR lower[Text Word] OR less[Text Word] OR least[Text Word] OR under[Text Word] OR mid[Text Word] OR middle[Text Word] OR third[Text Word])) AND (countr* or nation or econom*)	547054
#72	Search (LMIC[Text Word] OR LMICs[Text Word] OR LAMIC[Text Word] OR LAMICs[Text Word])	1833
#71	Search (#69) OR #70	40557
#70	Search (Tegucigalpa[Text Word] OR Tegus[Text Word] OR New Delhi[Text Word] OR Jakarta[Text Word] OR Nairobi[Text Word] OR South Tarawa[Text Word] OR Tarawa Teinainano[Text Word] OR Pristina[Text Word] OR Prishtina[Text Word] OR Bishkek[Text Word] OR Pishpek[Text Word] OR Frunze[Text Word] OR Vientiane[Text Word] OR Maseru[Text Word] OR Nouakchott[Text Word] OR Palikir[Text Word] OR Chisinau[Text Word] OR Kishinev[Text Word] OR Rabat[Text Word] OR Nay Pyi Taw[Text Word] OR Naypyidaw[Text Word] OR Nepranytau[Text Word] OR Naypyitaw[Text Word] OR Kyetpyay[Text Word] OR Pyinmana[Text Word] OR Kyatpyay[Text Word] OR Pyinmana[Text Word] OR Yangon[Text Word] OR Rangoon[Text Word] OR Managua[Text Word] OR Abuja[Text Word] OR Lagos[Text Word] OR Islamabad[Text Word] OR Port Moresby[Text Word] OR Moresby[Text Word] OR Pom Town[Text Word] OR Manila[Text Word] OR Apia[Text Word] OR Dakar[Text Word] OR Honiara[Text Word] OR Jayawardenepura[Text Word] OR Jayewardenepura[Text Word] OR Khartoum[Text Word] OR Mbabane[Text Word] OR Embabane[Text Word] OR Lobamba[Text Word] OR Damascus[Text Word] OR Dushanbe[Text Word] OR Dyushambe[Text Word] OR Stalinabad[Text Word] OR Dili[Text Word] OR Kyiv[Text Word] OR Kiev[Text Word] OR Tashkent[Text Word] OR Toshkent[Text Word] OR Port Vila[Text Word] OR Hanoi[Text Word] OR Ha Noi[Text Word] OR Sana'a[Text Word] OR Sanaa[Text Word] OR Sana[Text Word] OR Lusaka[Text Word] OR Ulaanbaatar[Text Word] OR Ulan-Bator[Text Word] OR Nuku-Alofa[Text Word])	18925
#69	Search (kabul[Text Word] OR Porto-Novo[Text Word] OR Hogbonou[Text Word] OR Adjace[Text Word] OR Cotonou[Text Word] OR Kutonu[Text	21867

tara.landry@muhc.mcgill.ca
http://www.muhclibraries.ca/

	Word] OR Ouagadougou[Text Word] OR Ouaga[Text Word] OR Bujumbura[Text Word] OR Usumbura[Text Word] OR Phnom Penh[Text Word] OR Bangui[Text Word] OR Bangi[Text Word] OR N'Djamena[Text Word] OR Ndjamena[Text Word] OR Fort Lamy[Text Word] OR Moroni[Text Word] OR Kinshasa[Text Word] OR Asmara[Text Word] OR Asmera[Text Word] OR Addis Ababa[Text Word] OR Addis Abeba[Text Word] OR Banjul[Text Word] OR Bathurst[Text Word] OR Conakry[Text Word] OR Bissau[Text Word] OR Port-au-Prince[Text Word] OR Pyongyang[Text Word] OR Monrovia[Text Word] OR Antananarivo[Text Word] OR Tananarive[Text Word] OR Tana[Text Word] OR Lilongwe[Text Word] OR Bamako[Text Word] OR Maputo[Text Word] OR Lourenco Marques[Text Word] OR Kathmandu[Text Word] OR Niamey[Text Word] OR Kigali[Text Word] OR Freetown[Text Word] OR Free-town[Text Word] OR Mogadishu[Text Word] OR Xamar[Text Word] OR Hamar[Text Word] OR Muqdisho[Text Word] OR Maqadishu[Text Word] OR Juba[Text Word] OR Dodoma[Text Word] OR Dar es Salaam[Text Word] OR Lome[Text Word] OR Kampala[Text Word] OR Harare[Text Word] OR Salisbury[Text Word] OR Yerevan[Text Word] OR Dhaka[Text Word] OR Dacca[Text Word] OR Thimphu[Text Word] OR Thimbu[Text Word] OR Sucre[Text Word] OR Charcas[Text Word] OR La Plata[Text Word] OR Chuquisaca[Text Word] OR La Paz[Text Word] OR Praia[Text Word] OR Yaounde[Text Word] OR Jaunde[Text Word] OR Brazzaville[Text Word] OR Yamoussoukro[Text Word] OR Cairo[Text Word] OR Accra[Text Word])	
#68	Search #3 OR #4 OR #5 OR #6 OR #7 OR #9 OR #9 OR #10 OR #11 OR #12 OR #13 OR #14 OR #15 OR #16 OR #17 OR #18 OR #19 OR #20 OR #21 OR #22 OR #23 OR #24 OR #25 OR #26 OR #27 OR #28 OR #30 OR #31 OR #32 OR #33 OR #34 OR #35 OR #36 OR #37 OR #38 OR #39 OR #40 OR #41 OR #42 OR #43 OR #44 OR #45 OR #46 OR #47 OR #48 OR #49 OR #50 OR #51 OR #52 OR #53 OR #54 OR #55 OR #56 OR #57 OR #58 OR #59 OR #60 OR #61 OR #62 OR #63 OR #64 OR #65 OR #66 OR #67	704488
#67	Search (zimbabwe[Text Word] OR rhodesia[Text Word])	6530
#66	Search zambia[Text Word]	4870
#65	Search (Yugoslavia[Text Word] OR vojvodina[Text Word])	10059
#64	Search (yemen*[Text Word] OR aden[Text Word] OR sanaa[Text Word])	2161
#63	Search (west bank[Text Word] OR gaza[Text Word] OR palestin*[Text Word])	2685
#62	Search (vietnam[Text Word] OR viet-nam[Text Word])	14854
#61	Search (vanuatu[Text Word] OR new hebrides[Text Word])	592
#60	Search uzbek*[Text Word]	2197
#59	Search ukrain*[Text Word]	17108
#58	Search (Tunisia[Text Word] OR Tunis[Text Word])	8634
#57	Search tonga*[Text Word]	612

#56	Search (Timor Leste[Text Word] OR East Timor[Text Word] OR Portuguese-Timor)	376
#55	Search (Tajikistan[Text Word] OR Tadjik*[Text Word] OR Tadzhik*[Text Word] OR Tajik[Text Word])	918
#54	Search syria*[Text Word]	10985
#53	Search swaziland[Text Word]	715
#52	Search (ceylon[Text Word] OR sri lanka[Text Word])	6755
#51	Search ((Caroline[Text Word] OR Ellice[Text Word] OR Gilbert[Text Word] OR Johnston[Text Word] OR Mariana[Text Word] OR Marshall[Text Word] OR Pacific[Text Word] OR Solomon[Text Word])) AND (Island[Text Word] OR islands[Text Word])	7519
#50	Search Sao Tome[Text Word]	201
#49	Search samoa*[Text Word]	1210
#48	Search (Philippines[Text Word] OR Philipines[Text Word] OR Phillipines[Text Word] OR Phillippines[Text Word] OR Mindanao[Text Word] OR Luzon[Text Word] OR Visayas[Text Word])	10061
#47	Search Papua[Text Word]	5217
#46	Search Pakistan[Text Word]	17935
#45	Search nicaragua[Text Word]	1799
#44	Search Nepal[Text Word]	8259
#43	Search (myanmar[Text Word] OR burma[Text Word])	3003
#42	Search (mozambique[Text Word] OR Mocambique[Text Word] OR mozambic[Text Word])	2899
#41	Search (morocco[Text Word] OR ifni[Text Word])	6246
#40	Search mongolia*[Text Word]	7437
#39	Search (moldova*[Text Word] OR Moldavia*[Text Word])	1023
#38	Search micronesia*[Text Word]	1537
#37	Search (malawi[Text Word] OR nyasaland)	6501
#36	Search (madagascar[Text Word] OR malagasy[Text Word])	4481
#35	Search (lesotho[Text Word] OR Basutoland)	618
#34	Search (lao[Text Word]) AND democratic republic[Text Word]	344
#33	Search (laos[Text Word] OR lao pdr[Text Word])	2331
#32	Search (Kirghiz*[Text Word] OR Kirgiz*[Text Word] OR Kyrgyz*[Text Word])	1507
#31	Search kosovo[Text Word]	743

#30	Search Korea[Text Word]	47383
#28	Search (kiribati[Text Word] OR nauru[Text Word] OR tuvalu[Text Word])	271
#27	Search Kashmir[Text Word]	1126
#26	Search (indonesia*[Text Word] OR Celebes[Text Word] OR East Indies[Text Word] OR Irian Jaya[Text Word] OR Java[Text Word] OR Madoera[Text Word] OR Madura[Text Word] OR Malay Archipelago[Text Word] OR "Netherlands East Indies"[Text Word] OR New Guinea West[Text Word] OR Sulawesi[Text Word] OR Sumatra[Text Word] OR Timor[Text Word] OR West Irian[Text Word])	16339
#25	Search (india[Text Word] OR Sikkim[Text Word])	118053
#24	Search honduras[Text Word]	1580
#23	Search (haiti[Text Word] OR hispaniola[Text Word])	3529
#22	Search Guatemala[Text Word]	3634
#21	Search salvador[Text Word]	2853
#20	Search (egypt[Text Word] OR "united arab republic"[Text Word])	17267
#19	Search (katanga[Text Word] OR zaire[Text Word] OR congo[Text Word] OR leopoldville[Text Word])	13026
#18	Search (comoros[Text Word] OR comores[Text Word] OR comoro[Text Word] OR mayotte[Text Word])	507
#17	Search (chad[Text Word] OR tchad[Text Word] OR tshad[Text Word])	1113
#16	Search ((central-africa*[Text Word] OR centrafrican[Text Word] OR central african[Text Word])) AND (republic[Text Word] OR empire[Text Word])	1354
#15	Search ("ubangi-shari"[Text Word] OR Centrafrique[Text Word])	2
#14	Search (cameroun[Text Word] OR cameroon[Text Word])	6212
#13	Search (cambodia[Text Word] OR Kampuchea*[Text Word] OR Khmer[Text Word] OR kampudja[Text Word])	3772
#12	Search boliv*[Text Word]	4178
#11	Search Bhutan[Text Word]	499
#10	Search (Bangladesh[Text Word] OR East-bengal[Text Word])	11499
#9	Search armenia*[Text Word]	2290
#8	Search (sudan*[Text Word]) AND republic[Text Word]	259
#7	Search (West*-Africa[Text Word] OR Benin[Text Word] OR Dahomey[Text Word] OR Burkina Faso[Text Word] OR Upper Volta[Text Word] OR Cape verde[Text Word] OR Cabo Verde[Text Word] OR cap-vert[Text Word] OR cote d'ivoire[Text Word] OR ivory coast[Text Word] OR Cote dilvoire[Text Word] OR Gambia[Text Word] OR Ghana[Text Word] OR Gold Coast[Text	232729

tara.landry@muhc.mcgill.ca
http://www.muhclibraries.ca/

	Word] OR Guinea[Text Word] OR Liberia[Text Word] OR Mali[Text Word] OR Mauritania[Text Word] OR Mauretania[Text Word] OR Niger[Text Word] OR Nigeria[Text Word] OR Senegal[Text Word] OR Senegambia[Text Word] OR Sierra Leone[Text Word] OR Togo[Text Word] OR Togolese[Text Word] OR Togoland)	
#6	Search (Somali*[Text Word]) AND (democratic[Text Word] OR republic[Text Word])	87
#5	Search (East* Africa[Text Word] OR Burundi[Text Word] OR Djibouti[Text Word] OR Somaliland[Text Word] OR Eritrea[Text Word] OR Ethiopia[Text Word] OR Abyssinia[Text Word] OR Kenya[Text Word] OR Rwanda[Text Word] OR Ruanda[Text Word] OR Somalia[Text Word] OR Sudan[Text Word] OR Tanzania[Text Word] OR Tanganyika[Text Word] OR Zanzibar[Text Word] OR Urundi[Text Word] OR Uganda[Text Word])	75694
#4	Search Afghanistan[Text Word]	5440
#3	Search (trauma[Text Word]) AND (database*[Text Word] OR data-base*[Text Word] OR regist*[Text Word])	12836

2.3. Ovid Embase

1 registration/ (54932)
2 disease registry/ (10168)
3 registry/ (145878)
4 data base/ (335295)
5 or/1-4 (513744)
6 exp injury/ (2191830)
7 emergency health service/ (91437)
8 6 or 7 (2259256)
9 5 and 8 (52056)
10 (trauma adj2 (database* or data-base* or regist*)).tw,kw. (4481)
11 9 or 10 (53060)
12 Afghanistan/ or afghanistan.tw,kw,in. (7072)
13 Armenia/ or armenia*.tw,kw,in. (6814)
14 Bangladesh/ or bangladesh.tw,kw,in. (23520)
15 Benin/ or (benin or dahomey).tw,kw,in. (8081)
16 Bhutan/ or Bhutan.tw,kw,in. (706)
17 Bolivia/ or bolivia.tw,kw,in. (5024)
18 burkina faso/ or (burkina faso or upper volta).tw,kw,in. (6194)
19 burundi/ or (burundi or urundi).tw,kw,in. (1099)
20 Cape verde/ or (Cape verde or Cabo Verde).tw,kw,in. (560)
21 Cambodia/ or (cambodia or Kampuchea* or Khmer or kampudja).tw,kw,in. (5467)
22 cameroon/ or (cameroon or cameroun).tw,kw,in. (11067)
23 central african republic/ or ("ubangi-shari" or Centrafrique or ((central-africa* or centrafrican or central african) adj2 (republic or empire))).tw,kw,in. (1196)
24 chad/ or (chad or tchad or tshad).tw,kw,in. (1952)
25 Comoros/ or (comoro* or comores or mayotte).tw,kw,in. (786)
26 Democratic Republic Congo/ or Congo/ or (brazzaville or kinshasa or katanga or zaire or congo or leopoldville).tw,kw,in. (18488)
27 cote d'ivoire/ or (cote d'ivoire or ivory coast or Cote dilvoire).tw,kw,in. (6639)
28 Djibouti/ or Djibouti.tw,kw,in. (525)
29 Egypt/ or (egypt or "united arab republic").tw,kw,in. (95406)
30 El Salvador/ or (salvador or el salvador).tw,kw,in. (24443)
31 eritrea/ or Eritrea.tw,kw,in. (3313)
32 Ethiopia/ or (Ethiopia or Abyssinia).tw,kw,in. (15681)
33 gambia/ or gambia.tw,kw,in. (4468)
34 Ghana/ or (ghana or gold coast).tw,kw,in. (20170)
35 Guatemala/ or Guatemala.tw,kw,in. (6067)
36 Guinea/ or Guinea.tw,kw,in. (141931)
37 Guinea-Bissau/ (1019)
38 Haiti/ or (haiti or hispaniola).tw,kw,in. (4323)
39 Honduras/ or honduras.tw,kw,in. (2439)
40 exp India/ or (india or Sikkim).tw,kw,in. (673495)
41 Indonesia/ or (indonesia* or Celebes or East Indies or Irian Jaya or Java or Madoera or Madura or Malay Archipelago or "Netherlands East Indies" or New Guinea West or Sulawesi or Sumatra or Timor or West Irian).tw,kw,in. (33232)

42 (kashmir or cashmere).tw,kw,in. (5343)
43 kenya/ or kenya.tw,kw,in. (30568)
44 kiribati/ or kiribati.tw,kw,in. (163)
45 North Korea/ or (North Korea or (Korea adj5 people's republic)).tw,kw,in. (1043)
46 Kosovo/ or kosovo.tw,kw,in. (1408)
47 Kyrgyzstan/ or (Kirghiz* or Kirgiz or Kyrgyz).tw,kw,in. (1941)
48 Laos/ or (laos or lao pdr or (lao and democratic republic)).tw,kw,in. (2997)
49 Lesotho/ or (lesotho or Basutoland).tw,kw,in. (801)
50 liberia/ or liberia.tw,kw,in. (2043)
51 Madagascar/ or (madagascar or Malagasy).tw,kw,in. (5837)
52 Malawi/ or malawi.tw,kw,in. (8114)
53 mali/ or mali.tw,kw,in. (6604)
54 Mauritania/ or (mauritania or mauretania).tw,kw,in. (804)
55 Federated States of Micronesia/ or micronesia.tw,kw,in. (1146)
56 Moldova/ or (moldova or Moldavia*).tw,kw,in. (2728)
57 Mongolia/ or Inner Mongolia/ or mongolia*.tw,kw,in. (13853)
58 Morocco/ or (morocco or ifni).tw,kw,in. (23719)
59 Mozambique/ or (mozambique or Mocambique or mozambic or "Portuguese East Africa").tw,kw,in. (4373)
60 Myanmar/ or (myanmar or burma).tw,kw,in. (4258)
61 Nepal/ or Nepal.tw,kw,in. (13919)
62 Nicaragua/ or nicaragua.tw,kw,in. (2548)
63 niger/ or niger.tw,kw,in. (15452)
64 nigeria/ or nigeria.tw,kw,in. (61654)
65 Pakistan/ or Pakistan.tw,kw,in. (58726)
66 Papua New Guinea/ or Papua New Guinea.tw,kw,in. (8084)
67 Philippines/ or (Philippines or Philipines or Phillipines or Phillippines or Mindanao or Luzon or Visayas).tw,kw,in. (20592)
68 rwanda/ or (Rwanda or Ruanda).tw,kw,in. (3839)
69 Samoa/ or samoa*.tw,kw,in. (1502)
70 "Sao Tome and Principe"/ or sao tome.tw,kw,in. (218)
71 senegal/ or (senegal or Senegambia).tw,kw,in. (11470)
72 sierra leone/ or sierra leone.tw,kw,in. (2447)
73 ((Caroline or Ellice or Gilbert or Johnston or Mariana or Marshall or Pacific or Solomon) adj1 (Island or islands)).tw,kw,in. (3952)
74 somalia/ or somalia.tw,kw,in. (2085)
75 sudan/ or sudan.tw,kw,in. (13584)
76 Sri Lanka/ or (sri lanka or ceylon).tw,kw,in. (12754)
77 Swaziland/ or swaziland.tw,kw,in. (1250)
78 Syrian Arab Republic/ or syria*.tw,kw,in. (15302)
79 Tajikistan/ or (Tajikistan or Tadjik* or Tadzhik* or tajik*).tw,kw,in. (3059)
80 tanzania/ or (Tanzania or Tanganyika or Zanzibar).tw,kw,in. (17598)
81 Timor-Leste/ or (Timor Leste or East Timor or Portuguese Timor).tw,kw,in. (566)
82 togo/ or (togo or togolese).tw,kw,in. (3469)
83 Tonga/ or tonga*.tw,kw,in. (1112)
84 Tunisia/ or (Tunisia or Tunis).tw,kw,in. (34817)
85 uganda/ or uganda.tw,kw,in. (20422)

86 Ukraine/ or ukrain*.tw,kw,in. (54023)
87 uzbekistan/ or uzbek*.tw,kw,in. (5387)
88 Vanuatu/ or (Vanuatu or new Hebrides).tw,kw,in. (710)
89 viet nam/ or (vietnam or viet nam).tw,kw,in. (23170)
90 palestine/ or (west bank or gaza or palestin*).tw,kw,in. (5699)
91 Yemen/ or (yemen* or aden or sana?a).tw,kw,in. (7276)
92 Zambia/ or zambia.tw,kw,in. (7442)
93 Zimbabwe/ or (zimbabwe or rhodesia or nyasaland).tw,kw,in. (10116)
94 Yugoslavia/ (9719)
95 limit 94 to yr="1994 - 2013" (2687)
96 or/12-93,95 (1562723)
97 (kabul or Porto-Novo or Hogbonou or Adjace or Cotonou or Kutonu or Ouagadougou or Ouaga or Bujumbura or Usumbura or Phnom Penh or Bangui or Bangi or N'Djamena or Ndjamena or Fort Lamy or Moroni or Kinshasa or Asmara or Asmera or Addis Ababa or Addis Abeba or Banjul or Bathurst or Conakry or Bissau or Port-au-Prince or Pyongyang or Monrovia or Antananarivo or Tananarive or Tana or Lilongwe or Bamako or Maputo or Lourenco Marques or Kathmandu or Niamey or Kigali or Freetown or Free-town or Mogadishu or Xamar or Hamar or Muqdisho or Maqadishu or Juba or Dodoma or Dar es Salaam or Lome or Kampala or Harare or Salisbury or Yerevan or Dhaka or Dacca or Thimphu or Thimbu or Sucre or Charcas or La Plata or Chuquisaca or La Paz or Praia or Yaounde or Jaunde or Brazzaville or Yamoussoukro or Cairo or Accra).tw,kw,in. (193523)
98 (Tegucigalpa or Tegus or New Delhi or Jakarta or Nairobi or South Tarawa or Tarawa Teinainano or Pristina or Prishtina or Bishkek or Pishpek or Frunze or Vientiane or Maseru or Nouakchott or Palikir or Chisinau or Kishinev or Rabat or Nay Pyi Taw or Naypyidaw or Nepranytau or Naypyitaw or Kyetpyay or Pyinmana or Kyatpyay or Pyinmana or Yangon or Rangoon or Managua or Abuja or Lagos or Islamabad or Port Moresby or Moresby or Pom Town or Manila or Apia or Dakar or Honiara or Jayawardenepura or Jayewardenepura or Khartoum or Mbabane or Embabane or Lobamba or Damascus or Dushanbe or Dyushambe or Stalinabad or Dili or Kyiv or Kiev or Tashkent or Toshkent or Port Vila or Hanoi or Ha Noi or Sana'a or Sanaa or Sana or Lusaka or Ulaanbaatar or Ulan-Bator or Nuku-Alofa).tw,kw,in. (216360)
99 97 or 98 (403715)
100 developing country/ (101086)
101 (LMIC or LMICs or LAMIC or LAMICs or ((deprived* or underserved or under-served or transitional or poor or underdevel* or devel* or low* or less or least or under or mid* or third) adj3 (countr* or nation or econom*))).tw,kw. (138689)
102 100 or 101 (188955)
103 96 or 99 or 102 (1762761)
104 11 and 103 (2804)
105 remove duplicates from 104 (2644)

tara.landry@muhc.mcgill.ca
http://www.muhclibraries.ca/

2.4. Africa-Wide Information

#	Query	Results
S67	S1 AND S66	62
S66	S61 OR S64 OR S65	1,558,344
S65	TX LMIC or LMICs or LAMIC or LAMICs or ((deprived* or underserved or under-served or transitional or poor or underdevel* or devel* or low* or less or least or under or mid* or third) N3 (countr* or nation or econom*))	187,408
S64	S62 OR S63	589,877
S63	TX Tegucigalpa or Tegus or New Delhi or Jakarta or Nairobi or South Tarawa or Tarawa Teinainano or Pristina or Prishtina or Bishkek or Pishpek or Frunze or Vientiane or Maseru or Nouakchott or Palikir or Chisinau or Kishinev or Rabat or Nay Pyi Taw or Naypyidaw or Nepranytau or Naypyitaw or Kyetpyay or Pyinmana or Kyatpyay or Pyinmana or Yangon or Rangoon or Managua or Abuja or Lagos or Islamabad or Port Moresby or Moresby or Pom Town or Manila or Apia or Dakar or Honiara or Jayawardenepura or Jayewardenepura or Khartoum or Mbabane or Embabane or Lobamba or Damascus or Dushanbe or Dyushambe or Stalinabad or Dili or Kyiv or Kiev or Tashkent or Toshkent or Port Vila or Hanoi or Ha Noi or Sana'a or Sanaa or Sana or Lusaka or Ulaanbaatar or Ulan-Bator or Nuku-Alofa	210,583
S62	TX kabul or Porto-Novo or Hogbonou or Adjace or Cotonou or Kutonu or Ouagadougou or Ouaga or Bujumbura or Usumbura or Phnom Penh or Bangui or Bangi or N'Djamena or Ndjamena or Fort Lamy or Moroni or Kinshasa or Asmara or Asmera or Addis Ababa or Addis Abeba or Banjul or Bathurst or Conakry or Bissau or Port-au-Prince or Pyongyang or Monrovia or Antananarivo or Tananarive or Tana or Lilongwe or Bamako or Maputo or Lourenco Marques or Kathmandu or Niamey or Kigali or Freetown or Free-town or Mogadishu or Xamar or Hamar or Muqdisho or Maqadishu or Juba or Dodoma or Dar es Salaam or Lome or Kampala or Harare or Salisbury or Yerevan or Dhaka or Dacca or Thimphu or Thimbu or Sucre or Charcas or La Plata or Chuquisaca or La Paz or Praia or Yaounde or Jaunde or Brazzaville or Yamoussoukro or Cairo or Accra	418,662
S61	S2 OR S3 OR S4 OR S5 OR S6 OR S7 OR S8 OR S9 OR S10 OR S11 OR S12 OR S13 OR S14 OR S15 OR S16 OR S17 OR S18 OR S19 OR S20 OR S21 OR S22 OR S23 OR S24 OR S25 OR S26 OR S27 OR S28 OR S29 OR S30 OR S31 OR S32 OR S33 OR S34 OR S35 OR S36 OR S37 OR S38 OR S39 OR S40 OR S41 OR S42 OR S43 OR S44 OR S45 OR S46 OR S47 OR S48 OR S49 OR S50 OR S51 OR S52 OR S53 OR S54 OR S55 OR S56 OR S57 OR S58 OR S59 OR S60	1,427,580
S60	TX zimbabwe or rhodesia	118,682
S59	TX zambia	35,950
S58	TX Yugoslavia or vojvodina	1,736

S57	TX yemen* or aden or sanaa	12,141
S56	TX west bank or gaza or palestin*	60,085
S55	TX vietnam or viet-nam	7,078
S54	TX vanuatu or new hebrides	774
S53	TX uzbek*	1,062
S52	TX ukrain*	3,986
S51	TX Tunisia or Tunis	43,149
S50	TX tonga*	2,100
S49	TX Timor Leste or East Timor or Portuguese-Timor	938
S48	TX Tajikistan or Tadjik* or Tadzhik* or Tajik	851
S47	TX syria*	28,866
S46	TX swaziland	13,496
S45	TX ceylon or sri lanka	5,020
S44	TX (Caroline or Ellice or Gilbert or Johnston or Mariana or Marshall or Pacific or Solomon) N1 (Island or islands)	2,246
S43	TX Sao Tome	4,400
S42	TX samoa*	598
S41	TX Philippines or Philipines or Phillipines or Phillippines or Mindanao or Luzon or Visayas	8,915
S40	TX Papua	5,386
S39	TX Pakistan	18,571
S38	TX nicaragua	1,612
S37	TX Nepal	3,863
S36	TX myanmar or burma	3,484
S35	TX mozambique or Mocambique or mozambic	35,127
S34	TX morocco or ifni	58,631
S33	TX mongolia*	3,260
S32	TX moldova* or Moldavia*	438
S31	TX micronesia*	359
S30	TX malawi or nyasaland	26,931
S29	TX madagascar or malagasy	30,926
S28	TX lesotho or Basutoland	16,343
S27	TX laos or lao pdr or (lao and democratic republic)	2,236
S26	TX Kirghiz* or Kirgiz* or Kyrgyz*	664
S25	TX kosovo	1,328
S24	TX North* Korea or (Korea adj5 demo*)	3,378
S23	TX kiribati or nauru or tuvalu	403
S22	TX Kashmir	1,117
S21	TX indonesia* or Celebes or East Indies or Irian Jaya or Java or Madoera or Madura or Malay Archipelago or "Netherlands East Indies" or New Guinea West	17,839

tara.landry@muhc.mcgill.ca
http://www.muhclibraries.ca/

	or Sulawesi or Sumatra or Timor or West Irian	
S20	TX india or Sikkim	68,207
S19	TX honduras	1,091
S18	TX haiti or hispaniola	3,192
S17	TX Guatemala	2,061
S16	TX salvador	2,431
S15	TX egypt or "united arab republic"	203,015
S14	TX katanga or zaire or congo or leopoldville	62,825
S13	TX comoros or comores or comoro or mayotte	4,062
S12	TX chad or tchad or tshad	21,204
S11	TX "ubangi-shari" or Centrafrique or ((central-africa* or centrafrican or central african) N2 (republic or empire))	9,234
S10	TX cameroun or cameroon	33,873
S9	TX cambodia or Kampuchea* or Khmer or kampudja	4,184
S8	TX boliv*	3,174
S7	TX Bhutan	374
S6	TX Bangladesh or East-bengal	6,708
S5	TX armenia*	1,723
S4	TX West*-Africa or Benin or Dahomey or Burkina Faso or Upper Volta or Cape verde or Cabo Verde or cap-vert or cote d'ivoire or ivory coast or Cote dilvoire or Gambia or Ghana or Gold Coast or Guinea or Liberia or Mali or Mauritania or Mauretania or Niger or Nigeria or Senegal or Senegambia or Sierra Leone or Togo or Togolese or Togoland or (sudan* N1 republic)	504,248
S3	TX East* Africa or Burundi or Djibouti or Somaliland or Eritrea or Ethiopia or Abyssinia or Kenya or Rwanda or Ruanda or Somalia or Sudan or Tanzania or Tanganyika or Zanzibar or Urundi or Uganda or (Somali* N1 (democratic or republic))	420,964
S2	TX Afghanistan	10,593
S1	TX trauma N2 (database* or data-base* or regist*)	180

2.5. Biosis Previews

1 (trauma adj2 (database* or data-base* or regist*)).ti,ab,mi. (1347)
2 afghanistan.ti,ab,mi,ge,cy. (4164)
3 (East* Africa or Burundi or Djibouti or Somaliland or Eritrea or Ethiopia or Abyssinia or Kenya or Rwanda or Ruanda or Somalia or Sudan or Tanzania or Tanganyika or Zanzibar or Urundi or Uganda or (Somali* adj1 (democratic or republic))).ti,ab,mi,ge,cy. (83718)
4 (West*-Africa or Benin or Dahomey or Burkina Faso or Upper Volta or Cape verde or Cabo Verde or cap-vert or cote d'ivoire or ivory coast or Cote dilvoire or Gambia or Ghana or Gold Coast or Guinea or Liberia or Mali or Mauritania or Mauretania or Niger or Nigeria or Senegal or Senegambia or Sierra Leone or Togo or Togolese or Togoland or (sudan* adj1 republic)).ti,ab,mi,ge,cy. (265352)
5 armenia*.ti,ab,mi,ge,cy. (7194)
6 (Bangladesh or East-bengal).ti,ab,mi,ge,cy. (17875)
7 Bhutan.ti,ab,mi,ge,cy. (1282)
8 boliv*.ti,ab,mi,ge,cy. (12238)
9 (cambodia or Kampuchea* or Khmer or kampudja).ti,ab,mi,ge,cy. (3754)
10 (cameroun or cameroon).ti,ab,mi,ge,cy. (9865)
11 ("ubangi-shari" or Centrafrique or ((central-africa* or centrafrican or central african) adj2 (republic or empire))).ti,ab,mi,ge,cy. (1178)
12 (chad or tchad or tshad).ti,ab,mi,ge,cy. (1433)
13 (comoros or comores or comoro or mayotte).ti,ab,mi,ge,cy. (1203)
14 (katanga or zaire or congo or leopoldville).ti,ab,mi,ge,cy. (14394)
15 (egypt or "united arab republic").ti,ab,mi,ge,cy. (62893)
16 salvador.ti,ab,mi,ge,cy. (2619)
17 Guatemala.ti,ab,mi,ge,cy. (5869)
18 (haiti or hispaniola).ti,ab,mi,ge,cy. (3198)
19 honduras.ti,ab,mi,ge,cy. (3179)
20 (india or Sikkim).ti,ab,mi,ge,cy. (563475)
21 (indonesia* or Celebes or East Indies or Irian Jaya or Java or Madoera or Madura or Malay Archipelago or "Netherlands East Indies" or New Guinea West or Sulawesi or Sumatra or Timor or West Irian).ti,ab,mi,ge,cy. (32535)
22 Kashmir.ti,ab,mi,ge,cy. (3174)
23 (kiribati or nauru or tuvalu).ti,ab,mi,ge,cy. (405)
24 (North* Korea or (Korea adj5 demo*)).ti,ab,mi,ge,cy. (3953)
25 kosovo.ti,ab,mi,ge,cy. (560)
26 (Kirghiz* or Kirgiz* or Kyrgyz*).ti,ab,mi,ge,cy. (2213)
27 (laos or lao pdr or (lao and democratic republic)).ti,ab,mi,ge,cy. (3662)
28 (lesotho or Basutoland).ti,ab,mi,ge,cy. (909)
29 (madagascar or malagasy).ti,ab,mi,ge,cy. (14694)
30 (malawi or nyasaland).ti,ab,mi,ge,cy. (6018)
31 micronesia*.ti,ab,mi,ge,cy. (1603)
32 (moldova* or Moldavia*).ti,ab,mi,ge,cy. (2755)
33 mongolia*.ti,ab,mi,ge,cy. (16016)
34 (morocco or ifni).ti,ab,mi,ge,cy. (15059)
35 (mozambique or Mocambique or mozambic).ti,ab,mi,ge,cy. (4802)
36 (myanmar or burma).ti,ab,mi,ge,cy. (5665)

37 Nepal.ti,ab,mi,ge,cy. (9885)
38 nicaragua.ti,ab,mi,ge,cy. (2793)
39 Pakistan.ti,ab,mi,ge,cy. (46781)
40 Papua.ti,ab,mi,ge,cy. (8711)
41 (Philippines or Philipines or Phillipines or Phillippines or Mindanao or Luzon or Visayas).ti,ab,mi,ge,cy. (21264)
42 samoa*.ti,ab,mi,ge,cy. (1800)
43 Sao Tome.ti,ab,mi,ge,cy. (528)
44 ((Caroline or Ellice or Gilbert or Johnston or Mariana or Marshall or Pacific or Solomon) adj1 (Island or islands)).ti,ab,mi,ge,cy. (5634)
45 (ceylon or sri lanka).ti,ab,mi,ge,cy. (11736)
46 swaziland.ti,ab,mi,ge,cy. (1074)
47 syria*.ti,ab,mi,ge,cy. (16384)
48 (Tajikistan or Tadjik* or Tadzhik* or Tajik).ti,ab,mi,ge,cy. (2987)
49 (Timor Leste or East Timor or Portuguese-Timor).ti,ab,mi,ge,cy. (307)
50 tonga*.ti,ab,mi,ge,cy. (1454)
51 (Tunisia or Tunis).ti,ab,mi,ge,cy. (21066)
52 ukrain*.ti,ab,mi,ge,cy. (41554)
53 uzbek*.ti,ab,mi,ge,cy. (7247)
54 (vanuatu or new hebrides).ti,ab,mi,ge,cy. (1475)
55 (vietnam or viet-nam).ti,ab,mi,ge,cy. (18990)
56 (west bank or gaza or palestin*).ti,ab,mi,ge,cy. (2018)
57 (yemen* or aden or sanaa).ti,ab,mi,ge,cy. (3152)
58 (Yugoslavia or vojvodina).ti,ab,mi,ge,cy. (31783)
59 zambia.ti,ab,mi,ge,cy. (5745)
60 (zimbabwe or rhodesia).ti,ab,mi,ge,cy. (9540)
61 or/2-60 (1347298)
62 (kabul or Porto-Novo or Hogbonou or Adjace or Cotonou or Kutonu or Ouagadougou or Ouaga or Bujumbura or Usumbura or Phnom Penh or Bangui or Bangi or N'Djamena or Ndjamena or Fort Lamy or Moroni or Kinshasa or Asmara or Asmera or Addis Ababa or Addis Abeba or Banjul or Bathurst or Conakry or Bissau or Port-au-Prince or Pyongyang or Monrovia or Antananarivo or Tananarive or Tana or Lilongwe or Bamako or Maputo or Lourenco Marques or Kathmandu or Niamey or Kigali or Freetown or Free-town or Mogadishu or Xamar or Hamar or Muqdisho or Maqadishu or Juba or Dodoma or Dar es Salaam or Lome or Kampala or Harare or Salisbury or Yerevan or Dhaka or Dacca or Thimphu or Thimbu or Sucre or Charcas or La Plata or Chuquisaca or La Paz or Praia or Yaounde or Jaunde or Brazzaville or Yamoussoukro or Cairo or Accra).ti,ab,mi,ge,cy. (18273)
63 (Tegucigalpa or Tegus or New Delhi or Jakarta or Nairobi or South Tarawa or Tarawa Teinainano or Pristina or Prishtina or Bishkek or Pishpek or Frunze or Vientiane or Maseru or Nouakchott or Palikir or Chisinau or Kishinev or Rabat or Nay Pyi Taw or Naypyidaw or Nepranytau or Naypyitaw or Kyetpyay or Pyinmana or Kyatpyay or Pyinmana or Yangon or Rangoon or Managua or Abuja or Lagos or Islamabad or Port Moresby or Moresby or Pom Town or Manila or Apia or Dakar or Honiara or Jayawardenepura or Jayewardenepura or Khartoum or Mbabane or Embabane or Lobamba or Damascus or Dushanbe or Dyushambe or Stalinabad or Dili or Kyiv or Kiev or Tashkent or Toshkent or Port Vila or Hanoi or Ha Noi or Sana'a or Sanaa or Sana or Lusaka or Ulaanbaatar or Ulan-Bator or Nuku-Alofa).ti,ab,mi,ge,cy. (17613)
64 62 or 63 (35690)

65 (LMIC or LMICs or LAMIC or LAMICs or ((deprived* or underserved or under-served or transitional or poor or underdevel* or devel* or low* or less or least or under or mid* or third) adj3 (countr* or nation or econom*))).ti,ab,mi. (67320)
66 61 or 64 or 65 (1403294)
67 1 and 66 (92)
68 remove duplicates from 67 (84)

2.6. The Cochrane Library

ID	SEARCH	RESULTS
#1	(trauma near/2 (database* or data-base* or regist*)):ti,ab,kw	58

2.7.

2.8. Global Health

1 (trauma adj2 (database* or data-base* or regist*)).ti,ab,id. (329)
2 Afghanistan.ti,ab,id,in,gl. (1701)
3 (East* Africa or Burundi or Djibouti or Somaliland or Eritrea or Ethiopia or Abyssinia or Kenya or Rwanda or Ruanda or Somalia or Sudan or Tanzania or Tanganyika or Zanzibar or Urundi or Uganda or (Somali* adj1 (democratic or republic))).ti,ab,id,in,gl. (48302)
4 (West*-Africa or Benin or Dahomey or Burkina Faso or Upper Volta or Cape verde or Cabo Verde or cap-vert or cote d'ivoire or ivory coast or Cote dilvoire or Gambia or Ghana or Gold Coast or Guinea or Liberia or Mali or Mauritania or Mauretania or Niger or Nigeria or Senegal or Senegambia or Sierra Leone or Togo or Togolese or Togoland or (sudan* adj1 republic)).ti,ab,id,in,gl. (81450)
5 armenia*.ti,ab,id,in,gl. (901)
6 (Bangladesh or East-bengal).ti,ab,id,in,gl. (11003)
7 Bhutan.ti,ab,id,in,gl. (386)
8 boliv*.ti,ab,id,in,gl. (3162)
9 (cambodia or Kampuchea* or Khmer or kampudja).ti,ab,id,in,gl. (2471)
10 (cameroun or cameroon).ti,ab,id,in,gl. (6359)
11 ("ubangi-shari" or Centrafrique or ((central-africa* or centrafrican or central african) adj2 (republic or empire))).ti,ab,id,in,gl. (903)
12 (chad or tchad or tshad).ti,ab,id,in,gl. (813)
13 (comoros or comores or comoro or mayotte).ti,ab,id,in,gl. (435)
14 (katanga or zaire or congo or leopoldville).ti,ab,id,in,gl. (7083)
15 (egypt or "united arab republic").ti,ab,id,in,gl. (23203)
16 salvador.ti,ab,id,in,gl. (4383)
17 Guatemala.ti,ab,id,in,gl. (2906)
18 (haiti or hispaniola).ti,ab,id,in,gl. (1681)
19 honduras.ti,ab,id,in,gl. (929)
20 (india or Sikkim).ti,ab,id,in,gl. (165425)
21 (indonesia* or Celebes or East Indies or Irian Jaya or Java or Madoera or Madura or Malay Archipelago or "Netherlands East Indies" or New Guinea West or Sulawesi or Sumatra or Timor or West Irian).ti,ab,id,in,gl. (9787)
22 Kashmir.ti,ab,id,in,gl. (1961)
23 (kiribati or nauru or tuvalu).ti,ab,id,in,gl. (194)
24 (North* Korea or (Korea adj5 demo*)).ti,ab,id,in,gl. (378)
25 kosovo.ti,ab,id,in,gl. (420)
26 (Kirghiz* or Kirgiz* or Kyrgyz*).ti,ab,id,in,gl. (483)
27 (laos or lao pdr or (lao and democratic republic)).ti,ab,id,in,gl. (1482)
28 (lesotho or Basutoland).ti,ab,id,in,gl. (487)
29 (madagascar or malagasy).ti,ab,id,in,gl. (2537)
30 (malawi or nyasaland).ti,ab,id,in,gl. (4613)
31 micronesia*.ti,ab,id,in,gl. (312)
32 (moldova* or Moldavia*).ti,ab,id,in,gl. (573)
33 mongolia*.ti,ab,id,in,gl. (3106)
34 (morocco or ifni).ti,ab,id,in,gl. (5205)
35 (mozambique or Mocambique or mozambic).ti,ab,id,in,gl. (2197)
36 (myanmar or burma).ti,ab,id,in,gl. (2716)

37 Nepal.ti,ab,id,in,gl. (5506)
38 nicaragua.ti,ab,id,in,gl. (1089)
39 Pakistan.ti,ab,id,in,gl. (18100)
40 Papua.ti,ab,id,in,gl. (3276)
41 (Philippines or Philipines or Phillipines or Phillippines or Mindanao or Luzon or Visayas).ti,ab,id,in,gl. (6071)
42 samoa*.ti,ab,id,in,gl. (575)
43 Sao Tome.ti,ab,id,in,gl. (137)
44 ((Caroline or Ellice or Gilbert or Johnston or Mariana or Marshall or Pacific or Solomon) adj1 (Island or islands)).ti,ab,id,in,gl. (1544)
45 (ceylon or sri lanka).ti,ab,id,in,gl. (4992)
46 swaziland.ti,ab,id,in,gl. (606)
47 syria*.ti,ab,id,in,gl. (2323)
48 (Tajikistan or Tadjik* or Tadzhik* or Tajik).ti,ab,id,in,gl. (523)
49 (Timor Leste or East Timor or Portuguese-Timor).ti,ab,id,in,gl. (253)
50 tonga*.ti,ab,id,in,gl. (337)
51 (Tunisia or Tunis).ti,ab,id,in,gl. (6416)
52 ukrain*.ti,ab,id,in,gl. (3799)
53 uzbek*.ti,ab,id,in,gl. (1773)
54 (vanuatu or new hebrides).ti,ab,id,in,gl. (423)
55 (vietnam or viet-nam).ti,ab,id,in,gl. (6964)
56 (west bank or gaza or palestin*).ti,ab,id,in,gl. (1318)
57 (yemen* or aden or sanaa).ti,ab,id,in,gl. (1557)
58 (Yugoslavia or vojvodina).ti,ab,id,in,gl. (3450)
59 zambia.ti,ab,id,in,gl. (3804)
60 (zimbabwe or rhodesia).ti,ab,id,in,gl. (4416)
61 or/2-60 (433104)
62 (kabul or Porto-Novo or Hogbonou or Adjace or Cotonou or Kutonu or Ouagadougou or Ouaga or Bujumbura or Usumbura or Phnom Penh or Bangui or Bangi or N'Djamena or Ndjamena or Fort Lamy or Moroni or Kinshasa or Asmara or Asmera or Addis Ababa or Addis Abeba or Banjul or Bathurst or Conakry or Bissau or Port-au-Prince or Pyongyang or Monrovia or Antananarivo or Tananarive or Tana or Lilongwe or Bamako or Maputo or Lourenco Marques or Kathmandu or Niamey or Kigali or Freetown or Free-town or Mogadishu or Xamar or Hamar or Muqdisho or Maqadishu or Juba or Dodoma or Dar es Salaam or Lome or Kampala or Harare or Salisbury or Yerevan or Dhaka or Dacca or Thimphu or Thimbu or Sucre or Charcas or La Plata or Chuquisaca or La Paz or Praia or Yaounde or Jaunde or Brazzaville or Yamoussoukro or Cairo or Accra).ti,ab,id,in,gl. (47191)
63 (Tegucigalpa or Tegus or New Delhi or Jakarta or Nairobi or South Tarawa or Tarawa Teinainano or Pristina or Prishtina or Bishkek or Pishpek or Frunze or Vientiane or Maseru or Nouakchott or Palikir or Chisinau or Kishinev or Rabat or Nay Pyi Taw or Naypyidaw or Nepranytau or Naypyitaw or Kyetpyay or Pyinmana or Kyatpyay or Pyinmana or Yangon or Rangoon or Managua or Abuja or Lagos or Islamabad or Port Moresby or Moresby or Pom Town or Manila or Apia or Dakar or Honiara or Jayawardenepura or Jayewardenepura or Khartoum or Mbabane or Embabane or Lobamba or Damascus or Dushanbe or Dyushambe or Stalinabad or Dili or Kyiv or Kiev or Tashkent or Toshkent or Port Vila or Hanoi or Ha Noi or Sana'a or Sanaa or Sana or Lusaka or Ulaanbaatar or Ulan-Bator or Nuku-Alofa).ti,ab,id,in,gl. (43135)
64 62 or 63 (89958)

65 (LMIC or LMICs or LAMIC or LAMICs or ((deprived* or underserved or under-served or transitional or poor or underdevel* or devel* or low* or less or least or under or mid* or third) adj3 (countr* or nation or econom*))).ti,ab,id. (54467)
66 61 or 64 or 65 (471660)
67 1 and 66 (67)
68 remove duplicates from 67 (67)

2.9. LILACS

tw:((tw:(trauma)) AND (tw:(register OR registers OR registry OR registries OR database OR databases OR data-base OR data-bases)) AND (instance:"regional")) AND (instance:"regional") AND (db:("LILACS" OR "IBECS" OR "BDENF" OR "CUMED" OR "MedCarib"))

2.10. ProQuest Dissertations & Theses Global

ti(trauma-regist* or trauma-database* or trauma-data-base*) OR ab(trauma-regist* or trauma-database* or trauma-data-base*)

2.11. Scopus

ID	SEARCH	HITS
#1	TITLE-ABS-KEY(trauma W/2 (database* OR data-base* OR regist*))	4,291
#2	TITLE-ABS-KEY(east* AND africa OR burundi OR djibouti OR somaliland OR eritrea OR ethiopia OR abyssinia OR kenya OR rwanda OR ruanda OR somalia OR sudan OR tanzania OR tanganyika OR zanzibar OR urundi OR uganda OR (somali* W/1 (democratic OR republic)))	66,274
#3	TITLE-ABS-KEY(west*-africa OR benin OR dahomey OR burkina AND faso OR upper AND volta OR cape AND verde OR cabo AND verde OR cap-vert OR cote AND d'ivoire OR ivory AND coast OR cote AND diivoire OR gambia OR ghana OR gold AND coast OR guinea OR liberia OR mali OR mauritania OR mauretania OR niger OR nigeria OR senegal OR senegambia OR sierra AND leone OR togo OR togolese OR togoland OR (sudan* W/1 republic))	9
#4	TITLE-ABS-KEY(afghanistan OR armenia* OR bangladesh OR east-bengal OR bhutan OR boliv* OR cambodia OR kampuchea* OR khmer OR kampudja OR cameroun OR cameroon OR "ubangi-shari" OR centrafrique)	95,203
#5	TITLE-ABS-KEY((centrafrican OR central-african)W/2 (republic OR empire))	3,239
#6	TITLE-ABS-KEY(central-africa* W/2 (republic OR empire))	447
#7	TITLE-ABS-KEY(chad OR tchad OR tshad OR comoros OR comores OR comoro OR mayotte OR katanga OR zaire OR congo OR leopoldville OR egypt OR "united arab republic" OR salvador OR guatemala OR haiti OR hispaniola OR honduras OR india OR sikkim OR indonesia* OR celebes OR east AND indies OR irian AND jaya OR java OR madoera OR madura OR malay AND archipelago OR "Netherlands East Indies" OR new AND guinea AND west OR sulawesi OR sumatra OR timor OR west AND irian)	66
#7	TITLE-ABS-KEY(kashmir OR kiribati OR nauru OR tuvalu OR north*-korea OR (korea W/5 demo*) OR kosovo OR kirghiz* OR kirgiz* OR kyrgyz* OR laos OR lao AND pdr OR (lao AND democratic-republic) OR lesotho OR basutoland OR madagascar OR malagasy OR malawi OR nyasaland OR micronesia*)	1,933
#8	TITLE-ABS-KEY(moldova* OR moldavia* OR mongolia* OR morocco OR ifni OR mozambique OR mocambique OR mozambic OR myanmar OR burma OR nepal OR nicaragua OR pakistan OR papua OR philippines OR philipines OR phillipines OR phillippines OR mindanao OR luzon OR visayas)	189,391
#9	TITLE-ABS-KEY(samoa* OR sao AND tome OR (caroline OR ellice OR gilbert OR johnston OR mariana OR marshall OR pacific OR solomon)W/1 (island OR islands) OR ceylon OR sri AND lanka OR swaziland OR syria* OR tajikistan OR tadjik* OR tadzhik* OR tajik OR timor AND leste OR east AND timor OR portuguese-timor OR tonga* OR tunisia OR tunis)	7
#10	TITLE-ABS-KEY(ukrain* OR uzbek* OR vanuatu OR new AND hebrides OR vietnam OR viet-nam OR west AND bank OR gaza OR palestin* OR yemen* OR aden OR	1,886

tara.landry@muhc.mcgill.ca
http://www.muhclibraries.ca/

	sanaa OR yugoslavia OR vojvodina OR zambia OR zimbabwe OR rhodesia)	
#11	#2 OR #3 OR #4 OR #5 OR #6 OR #7 OR #8 OR #9 OR #10	342,829
#12	TITLE-ABS-KEY(kabul OR porto-novo OR hogbonou OR adjace OR cotonou OR kutonu OR ouagadougou OR ouaga OR bujumbura OR usumbura OR phnom-penh OR bangui OR bangi OR n'djamena OR ndjamena OR fort-lamy OR moroni OR kinshasa OR asmara OR asmera OR addis-ababa OR addis-abeba OR banjul OR bathurst OR conakry OR bissau OR port-au-prince OR pyongyang OR monrovia OR antananarivo OR tananarive OR tana OR lilongwe OR bamako OR maputo OR lourenco-marques OR kathmandu OR niamey OR kigali OR freetown OR free-town OR mogadishu OR xamar OR hamar OR muqdisho OR maqadishu OR juba OR dodoma OR dar-es-salaam OR lome OR kampala OR harare OR salisbury OR yerevan OR dhaka OR dacca OR thimphu OR thimbu OR sucre OR charcas OR la-plata OR chuquisaca OR la-paz OR praia OR yaounde OR jaunde OR brazzaville OR yamoussoukro OR cairo OR accra OR tegucigalpa OR tegus OR new-delhi OR jakarta OR nairobi OR south-tarawa OR tarawa-teinainano OR pristina OR prishtina OR bishkek OR pishpek OR frunze OR vientiane OR maseru OR nouakchott OR palikir OR chisinau OR kishinev OR rabat OR nay-pyi-taw OR naypyidaw OR nepranytau OR naypyitaw OR kyetpyay OR pyinmana OR kyatpyay OR pyinmana OR yangon OR rangoon OR managua OR abuja OR lagos OR islamabad OR port-moresby OR moresby OR pom-town OR manila OR apia OR dakar OR honiara OR jayawardenepura OR jayewardenepura OR khartoum OR mbabane OR embabane OR lobamba OR damascus OR dushanbe OR dyushambe OR stalinabad OR dili OR kyiv OR kiev OR tashkent OR toshkent OR port-vila OR hanoi OR ha-noi OR sana'a OR sanaa OR sana OR lusaka OR ulaanbaatar OR ulan-bator OR nuku-alofa)	95,498
#13	TITLE-ABS-KEY(lmic OR lmics OR lamic OR lamics)	1,892
#14	TITLE-ABS-KEY((deprived* OR underserved OR under-served OR transitional OR poor OR underdevel* OR devel* OR low* OR less OR least OR under OR mid* OR third)W/3 (countr* OR nation OR econom*))	515,284
#15	#11 OR #12 OR #13 OR #14	889,779
#16	#1 AND #16	272

2.12. Web of Science

ID	SEARCH	HITS
# 15	#14 AND #1	251
# 14	#13 OR #12 OR #8	1,303,954
# 13	TS=(LMIC or LMICs or LAMIC or LAMICs or ((deprived* or underserved or under-served or transitional or poor or underdevel* or devel* or low* or less or least or under or mid* or third) NEAR/3 (countr* or nation or econom*)))	279,365
# 12	#11 OR #10	63,622
# 11	TS=(Tegucigalpa or Tegus or New-Delhi or Jakarta or Nairobi or South-Tarawa or Tarawa-Teinainano or Pristina or Prishtina or Bishkek or Pishpek or Frunze or Vientiane or Maseru or Nouakchott or Palikir or Chisinau or Kishinev or Rabat or Nay-Pyi-Taw or Naypyidaw or Nepranytau or Naypyitaw or Kyetpyay or Pyinmana or Kyatpyay or Pyinmana or Yangon or Rangoon or Managua or Abuja or Lagos or Islamabad or Port-Moresby or Moresby or Pom-Town or Manila or Apia or Dakar or Honiara or Jayawardenepura or Jayewardenepura or Khartoum or Mbabane or Embabane or Lobamba or Damascus or Dushanbe or Dyushambe or Stalinabad or Dili or Kyiv or Kiev or Tashkent or Toshkent or Port-Vila or Hanoi or Ha-Noi or Sana'a or Sanaa or Sana or Lusaka or Ulaanbaatar or Ulan-Bator or Nuku-Alofa)	29,417
# 10	TS=(kabul or Porto-Novo or Hogbonou or Adjace or Cotonou or Kutonu or Ouagadougou or Ouaga or Bujumbura or Usumbura or Phnom-Penh or Bangui or Bangi or N'Djamena or Ndjamena or Fort-Lamy or Moroni or Kinshasa or Asmara or Asmera or Addis-Ababa or Addis-Abeba or Banjul or Bathurst or Conakry or Bissau or Port-au-Prince or Pyongyang or Monrovia or Antananarivo or Tananarive or Tana or Lilongwe or Bamako or Maputo or Lourenco-Marques or Kathmandu or Niamey or Kigali or Freetown or Free-town or Mogadishu or Xamar or Hamar or Muqdisho or Maqadishu or Juba or Dodoma or Dar-es-Salaam or Lome or Kampala or Harare or Salisbury or Yerevan or Dhaka or Dacca or Thimphu or Thimbu or Sucre or Charcas or La-Plata or Chuquisaca or La-Paz or Praia or Yaounde or Jaunde or Brazzaville or Yamoussoukro or Cairo or Accra)	34,712
# 9	CI=(kabul or Porto-Novo or Hogbonou or Adjace or Cotonou or Kutonu or Ouagadougou or Ouaga or Bujumbura or Usumbura or Phnom-Penh or Bangui or Bangi or N'Djamena or Ndjamena or Fort-Lamy or Moroni or Kinshasa or Asmara or Asmera or Addis-Ababa or Addis-Abeba or Banjul or Bathurst or Conakry or Bissau or Port-au-Prince or Pyongyang or Monrovia or Antananarivo or Tananarive or Tana or Lilongwe or Bamako or Maputo or Lourenco-Marques or Kathmandu or Niamey or Kigali or Freetown or Free-town or Mogadishu or	265,806

	Xamar or Hamar or Muqdisho or Maqadishu or Juba or Dodoma or Dar-es-Salaam or Lome or Kampala or Harare or Salisbury or Yerevan or Dhaka or Dacca or Thimphu or Thimbu or Sucre or Charcas or La-Plata or Chuquisaca or La-Paz or Praia or Yaounde or Jaunde or Brazzaville or Yamoussoukro or Cairo or Accra)	
# 8	#7 OR #6 OR #5 OR #4 OR #3 OR #2	1,049,715
# 7	TS=(ukrain* or uzbek* or vanuatu or new-hebrides or vietnam or viet-nam or west-bank or gaza or palestin* or yemen* or aden or sanaa or Yugoslavia or vojvodina or zambia or zimbabwe or rhodesia)	105,676
# 6	TS=(samoa* or Sao-Tome or ((Caroline or Ellice or Gilbert or Johnston or Mariana or Marshall or Pacific or Solomon) NEAR/1 (Island or islands)) or ceylon or sri lanka or swaziland or syria* or Tajikistan or Tadjik* or Tadzhik* or Tajik or Timor Leste or East Timor or Portuguese-Timor or tonga* or Tunisia or Tunis)	66,298
# 5	TS=(moldova* or Moldavia* or mongolia* or morocco or ifni or mozambique or Mocambique or mozambic or myanmar or burma or Nepal or nicaragua or Pakistan or Papua or Philippines or Philipines or Phillipines or Phillippines or Mindanao or Luzon or Visayas)	137,752
# 4	TS=(Kashmir or kiribati or nauru or tuvalu or North*-Korea or (Korea NEAR/5 demo*) or kosovo or Kirghiz* or Kirgiz* or Kyrgyz* or laos or lao pdr or (lao and democratic-republic) or lesotho or Basutoland or madagascar or malagasy or malawi or nyasaland or micronesia*)	44,106
# 3	TS=(chad or tchad or tshad or comoros or comores or comoro or mayotte or katanga or zaire or congo or leopoldville or egypt or "united arab republic" or salvador or Guatemala or haiti or hispaniola or honduras or india or Sikkim or indonesia* or Celebes or East Indies or Irian Jaya or Java or Madoera or Madura or Malay-Archipelago or "Netherlands East Indies" or New-Guinea West or Sulawesi or Sumatra or Timor or West-Irian)	346,780
# 2	TS=(Afghanistan OR East*-Africa or Burundi or Djibouti or Somaliland or Eritrea or Ethiopia or Abyssinia or Kenya or Rwanda or Ruanda or Somalia or Sudan or Tanzania or Tanganyika or Zanzibar or Urundi or Uganda OR (Somali* NEAR/1 (democratic or republic)) OR West*-Africa or Benin or Dahomey or Burkina Faso or Upper Volta or Cape verde or Cabo Verde or cap-vert or cote d'ivoire or ivory coast or Cote dilvoire or Gambia or Ghana or Gold Coast or Guinea or Liberia or Mali or Mauritania or Mauretania or Niger or Nigeria or Senegal or Senegambia or Sierra Leone or Togo or Togolese or Togoland or (sudan* NEAR/1 republic) OR armenia* OR Bangladesh or East-bengal OR Bhutan OR boliv* OR cambodia or Kampuchea* or Khmer or kampudja OR cameroun or cameroon OR ubangi-shari	444,719

Tara Landry, MLIS
MUHC Montreal General Hospital Medical Library
tara.landry@muhc.mcgill.ca
http://www.muhclibraries.ca/

	or Centrafrique or ((central-africa* or centrafrican or central-african) NEAR/2 (republic or empire)))	
# 1	TS=(trauma NEAR/2 (database* or data-base* or regist*))	3,582

Tara Landry, MLIS
MUHC Montreal General Hospital Medical Library
tara.landry@muhc.mcgill.ca
http://www.muhclibraries.ca/
