# Supplementary material for: Strategies for successful trauma registry implementation in low- and middle-income countries—protocol for a systematic review
Source: Syst Rev. 2018 Feb 21;7:33. doi: 10.1186/s13643-018-0700-2 (PMC5822522; doi:10.1186/s13643-018-0700-2)
Supplement: Supplementary file 2 — Data Extraction Sheet. Template that will be used to extract data from included articles in systematic review. (PDF 51 kb) [file 13643_2018_700_MOESM2_ESM.pdf]

Additional File 2. Data extraction sheet Study #: ____

Title: ____
ID/DOI/URL: ____

Low- and middle-income country authorship (yes/no)	
LMIC authorship (position)	
Open access	
Study type	
Country of study	
World bank classification	
Number of patients	
Trauma centre designation	
Registry inclusion criteria	
Registry exclusion criteria	
Registry funding	
Source of funding	
High-income country partnership	
Registry modality	
Software used	
Platform compatibility	
Per-patient completion time	
Dedicated staff registry (yes/no)	
Data collectors	
Specialized training / data-collection certification	
Timing of data collection	
Vital signs	
Anatomical injury data	
Injury coding	
Severity of injury score (yes/no)	
Which score used?	
Patient demographics	
Procedures recorded?	
Complications recorded?	
Follow-up data?	
Type of follow-up data?	
Duration of follow-up?	
Data quality control?	
Challenges and barriers	
Successes and recommendations	
Comments	
